# Screening for Subtelomeric Rearrangements in Thai Patients with Intellectual Disabilities Using FISH and Review of Literature on Subtelomeric FISH in 15,591 Cases with Intellectual Disabilities

**DOI:** 10.1155/2016/9153740

**Published:** 2016-10-16

**Authors:** Chariyawan Charalsawadi, Jariya Khayman, Verayuth Praphanphoj, Pornprot Limprasert

**Affiliations:** ^1^Department of Pathology, Faculty of Medicine, Prince of Songkla University, Songkhla 90110, Thailand; ^2^Medical Genetics Center, Bangkok 10220, Thailand

## Abstract

We utilized fluorescence in situ hybridization (FISH) to screen for subtelomeric rearrangements in 82 Thai patients with unexplained intellectual disability (ID) and detected subtelomeric rearrangements in 5 patients. Here, we reported on a patient with der(20)t(X;20)(p22.3;q13.3) and a patient with der(3)t(X;3)(p22.3;p26.3). These rearrangements have never been described elsewhere. We also reported on a patient with der(10)t(7;10)(p22.3;q26.3), of which the same rearrangement had been reported in one literature. Well-recognized syndromes were detected in two separated patients, including 4p deletion syndrome and 1p36 deletion syndrome. All patients with subtelomeric rearrangements had both ID and multiple congenital anomalies (MCA) and/or dysmorphic features (DF), except the one with der(20)t(X;20), who had ID alone. By using FISH, the detection rate of subtelomeric rearrangements in patients with both ID and MCA/DF was 8.5%, compared to 2.9% of patients with only ID. Literature review found 28 studies on the detection of subtelomeric rearrangements by FISH in patients with ID. Combining data from these studies and our study, 15,591 patients were examined and 473 patients with subtelomeric rearrangements were determined. The frequency of subtelomeric rearrangements detected by FISH in patients with ID was 3%. Terminal deletions were found in 47.7%, while unbalanced derivative chromosomes were found in 47.9% of the rearrangements.

## 1. Introduction

An intellectual disability (ID) is a condition wherein the development of the mentality is arrested or incomplete, which contributes to the impairment of the overall level of intelligence, including cognitive, language, motor, and social abilities [1]. ID affects 1% to 3% of the global population. Besides environmental factors, genetic factors are a significant cause of ID. More than half of all patients with ID are categorized as having an unexplained ID, with subtelomeric rearrangements having been observed in a number of these patients, ranging between 0 and 29.4% [2].

The subtelomere is a region between chromosome-specific sequences and telomeric caps of each chromosome. This region is located in close proximity to a gene-rich area. Due to sequence homology between subtelomeres of different chromosomes, it can facilitate recombination and may subsequently result in detrimental effects, that is, promoting disease-causing chromosomal rearrangements [3].

Subtelomeric rearrangements can be detected by various methods. In this study, we utilized fluorescent in situ hybridization (FISH) method with probes specific to the subtelomere region of all chromosome ends to screen for subtelomeric rearrangements in patients with unexplained ID. We identified new rearrangements and described clinical entities of patients with the rearrangements.

## 2. Subjects and Methods

Inclusion criteria for subject recruitment were patients with ID of unknown causes, with or without multiple congenital anomalies (MCA) and/or dysmorphic features (DF) and with normal G-banding chromosome analysis result at the 450–550 bands' levels. All patients who met these inclusion criteria were referred from clinicians at Rajanukul Institute. The institute is the governmental agency under the Department of Mental Health, Ministry of Public Health, providing medical care primarily for individuals with intellectual and developmental disabilities. Clinical features of some patients may be limited as we collected data from the laboratory order form. A total of 82 Thai patients with ID were recruited to the study. These patients included 50 males and 32 females aged between 1 year and 39 years (mean age being 4 years). ID without MCA/DF was present in 35 cases (22 males and 13 females), and ID with MCA/DF was present in 47 cases (28 males and 19 females). This study was a one-year prospective study that was conducted between the years of 2005 and 2006.

We performed subtelomeric FISH analysis on metaphase spreads obtained from lymphocyte cultures, which were initiated and harvested following a standard protocol. The FISH probes used in this study were constructed using bacterial artificial chromosome (BAC) as well as P1-derived artificial chromosome (PAC) clones. These clones contained 41 different subtelomeric-specific sequences for all human chromosome ends, located within 2 Mb distance from the telomere. For the FISH analysis of each patient, the p-arm and q-arm probes of each chromosome were denatured and hybridized onto the denatured metaphase spreads. FISH was performed following a standard protocol. We examined at least 10 informative metaphase spreads for each chromosome. For patients with detected subtelomeric rearrangements, G-banding karyotype and FISH analyses using the same probes were carried out in parental blood when available. In addition, because the subtelomeric probe for the short arms of both X and Y chromosomes is specific to the pseudoautosomal region, the other X- and Y-specific probes were used to distinguish the chromosomes. This study was approved by the ethical committee of the Ministry of Public Health.

## 3. Results

**Figure 1:**
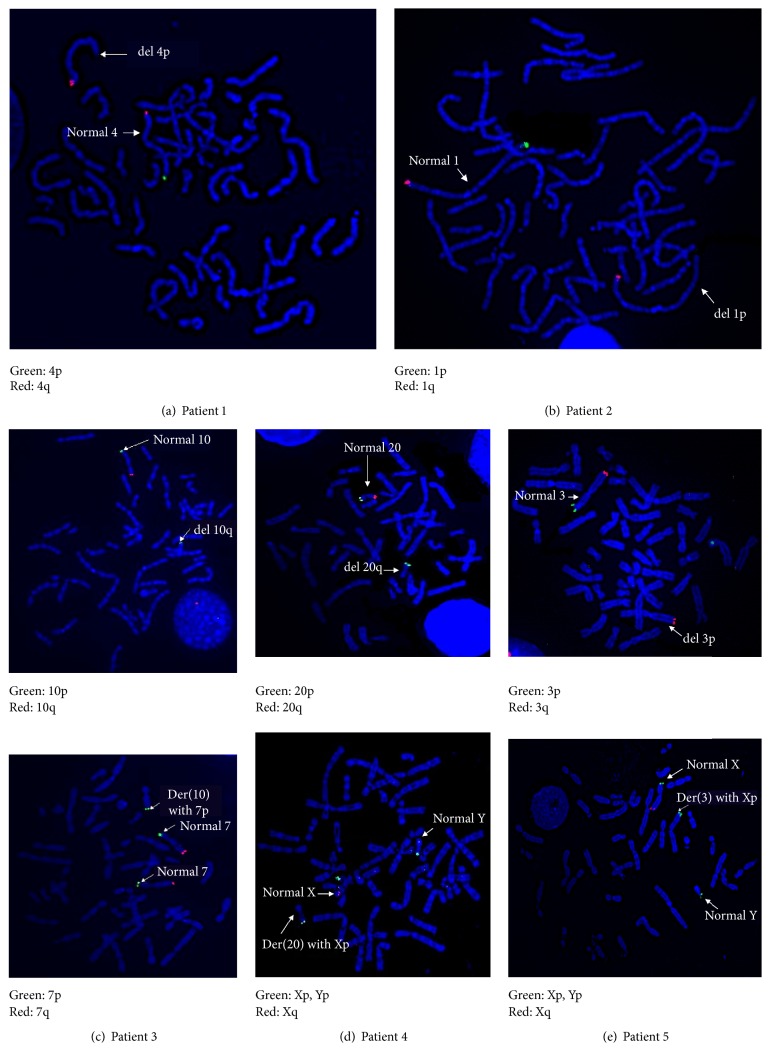
FISH results of 5 patients with subtelomeric rearrangements.

We detected 5 subtelomeric rearrangements in 5 patients. The frequency of subtelomeric rearrangements in our study was 6.1%. Two patients had deletions, including one with del(4)(p16.3) (Patient 1, Figure 1(a)) and one with del(1)(p36.3)dn (Patient 2, Figure 1(b)). The other three patients had derivative chromosomes, including one with der(10)t(7;10)(p22.3;q26.3) (Patient 3, Figure 1(c)), one with der(20)t(X;20)(p22.3;q13.3) (Patient 4, Figure 1(d)), and one with der(3)t(X;3)(p22.3;p26.3)dn (Patient 5, Figure 1(e)). Two patients had moderate degrees of ID, and 3 patients had severe degrees of ID, and those 3 patients included 2 patients with deletions. MCA/DF were observed in almost all of the patients with subtelomeric rearrangements, except a patient with der(20)t(X;20), who had only minor DF (Table 1). The frequency of subtelomeric rearrangements in patients with ID and MCA/DF was 8.5%, while in patients with only ID it was 2.9%.

## 4. Discussion

### 4.1. Detection of Subtelomeric Rearrangements

Subtelomeric rearrangements can be detected using various methods, such as FISH [4, 26], multiplex ligation-dependent probe amplification (MLPA) [32–34], and a microarray-based method [35, 36]. The latter method allows the whole human genome to be scanned at high resolution in a single experiment. As a result, not only cryptic subtelomeric rearrangements but also other unbalanced chromosomal abnormalities can be detected, such as interstitial deletions, duplications, and unbalanced translocations. However, FISH has the advantage of providing an instant location of certain rearrangements, such as insertions, inversions, and balanced translocations, which cannot be achieved by the microarray-based method.

We reviewed literature on subtelomeric rearrangements in individuals with ID, which were detected by subtelomeric FISH (Table 2). We search for articles in PubMed using the following keywords: subtelomeric FISH, intellectual disability, developmental delay, and mental retardation. We excluded studies from authors that did not provide details regarding number of the patients with ID, studies when FISH was performed to confirm findings of other molecular methods, studies where a selected panel of subtelomeric probes was used in each case, and extended studies with additional cases from the previous populations. We found 28 publications on the detection of subtelomeric rearrangements by FISH in patients with ID. Combining data from these studies (15,509 patients) and our study (82 patients), 15,591 patients with ID were examined by FISH and 474 subtelomeric rearrangements were identified in 473 patients. There was one patient with 2 rearrangements. Frequencies of subtelomeric rearrangements ranged from 0 to 20%, with an average of 3% (473/15,591) (Table 2). Familial variants and possible variants were found in approximately 1.0% (149/15,591) (Table 3). One of the most common variants is the del(2)(qter), in which the detection of a deletion depends on the subtelomeric probe used. In this literature review, we found that frequency of the del(2)(qter) was 42% of all variants (63/149). It is important to do parental analysis with the same subtelomeric probe when an abnormality is detected in a patient to determine the clinical significance of the finding.

**Figure 2:**
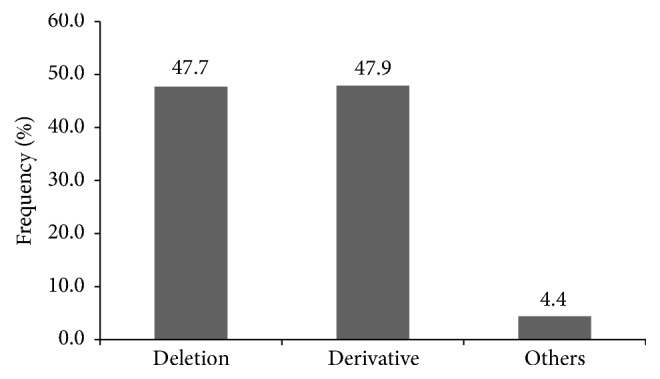
Frequency (%) of each category of subtelomeric rearrangements.

**Figure 3:**
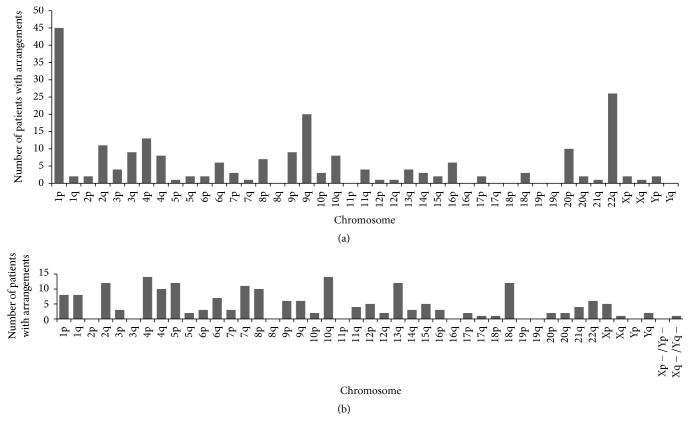
Number of patients with subtelomeric rearrangements involving each chromosome region. (a) Terminal deletion (*n* = 226). (b) Unbalanced derivative. The only chromosome in which the subtelomere region was monosomy is depicted here (*n* = 204).

We divided subtelomeric rearrangements into 3 categories: (1) deletion, (2) derivative, and (3) others. The latter category included duplications, insertions, inversions, isochromosome, and balanced translocations. Deletions were found in approximately 47.7% (226/474), while derivative chromosomes were found in approximately 47.9% (227/474) (Figure 2). The most frequent deletions (>5% of all deletions) involved chromosomes 1p, 22q, 9q, and 4p (Figure 3(a)). The derivative category was composed of an unbalanced translocation with both deletion and duplication of subtelomere regions, an unbalanced translocation with duplication of a subtelomere region onto the short arm of acrocentric chromosome, and an unbalanced translocation with only duplication or deletion of a subtelomere region detected. Monosomies associated with unbalanced derivative chromosomes frequently involved chromosomes 4p, 10q, 2q, 5p, 13q, 18q, and 7q (frequency >5% of all the monosomies) (Figure 3(b)). In this study, we detected subtelomeric rearrangements in 6.1% of the patients. Deletions of chromosomes 1p and 4p, which were among the most frequently detected deletions, were also detected in our study.

Attempts have been made to increase the diagnostic yield of the subtelomeric FISH method. A clinical checklist was developed to preselect patients for investigation. The selection criteria included prenatal/postnatal growth retardation, the presence of dysmorphism and/or congenital anomalies, and a family history of ID [37]. We found that frequency of subtelomeric rearrangements in patients with ID and MCA/DF was higher than that of those patients with ID alone (8.5% versus 2.9%). Our finding supported the suggestion that preselection of patients using the above-mentioned checklist, or a similar one, for subtelomeric FISH may increase the positive findings in patients with ID.

### 4.2. Clinical Features of Patients with Subtelomeric Rearrangements

In this study, we detected two well-recognized syndromes, namely, 4p deletion syndrome or Wolf-Hirschhorn syndrome and 1p36 deletion syndrome, in Patient 1 and Patient 2, respectively. Both syndromes have overlapping clinical features, including growth retardation, variable degree of ID/developmental delay, structural brain abnormalities, hypotonia, seizures, skeletal abnormalities, congenital heart defects, and hearing loss [38–40]. However, they are clinically recognized by distinct facial features, such as a Greek warrior helmet appearance of the nose, along with prominent glabella in the 4p deletion syndrome, and straight eyebrows, deeply set eyes, and midface retrusion in the 1p36 deletion syndrome. Clinical features of these syndromes may be variable depending on the extent of the deletions, in addition to the number and significance of the deleted genes. The deletions can be detected by using standard karyotype analysis in 50–60% of patients with 4p deletion syndrome and 25% of patients with 1p36 deletion syndrome, while FISH and chromosomal microarray (CMA) can detect chromosomal rearrangements in over 95% of the patients [38, 39]. In this study, our patients with 4p deletion and 1p36 deletion syndromes had subtle facial features, most likely because they had cryptic subtelomeric deletions.

Approximately half of the subtelomeric rearrangements detected in patients with ID were unbalanced derivative chromosomes. The presence of derivative chromosome results in deletion (i.e., partial monosomy) along with duplication (i.e., partial trisomy) of distinct subtelomere regions. Clinical features that presented in our patients with derivative chromosomes were influenced by the coexistence of two genomic imbalances, from which phenotypic consequence resulting from one type of genomic imbalance confounds the phenotypic consequence resulting from the other genomic imbalance. In addition, variable expressivity, incomplete penetrance, and the degree of skewed X-inactivation when X chromosome involved in the derivative chromosomes could have an influence on the phenotypes of the patients.

Patient 3 possessed der(10)t(7;10)(p22.3;q26.3), representing three 7p subtelomeres and only one 10q subtelomere. We found only one previous report of a patient with der(10)t(7;10)(p22.3;q26.3). She was a 17-year-old woman with short stature and moderate ID. Unlike our patient, she showed no dysmorphic facies and microcephaly [22]. Difference in clinical features may be due to the extent of the deletion and duplication. Duplication of 7p22.3 was reported on a patient with Asperger syndrome [41] and a patient with DF and skeletal abnormalities, including abnormal distal humeri [42]. A deformity of the elbow was present in our patient. In addition, developmental delay and minor DF were reported in the other patient with interstitial deletion of 10q26.3 [43].

Duplication of Xp22.3 that was detected in Patient 4 and Patient 5 was also detected in 2 out of 129 Thai patients with unexplained ID from a different cohort, who had subtelomeric rearrangements detected by MLPA technique [44]. Frequency of Xp22.3 duplication in Thai patients with unexplained ID was 1.9% (4/211). The duplication of Xp22.3 was observed in individuals with neurocognitive and behavioral abnormalities [45]. Moreover, deletion of Xp22.3 was associated with X-linked mental retardation and attention deficit hyperactivity disorder [45]. Patient 4 with deletion of 20q13.3 had only moderate ID with minor DF. Deletion of 20q13.33 was previously reported on patients with epileptic seizures. For Patient 5, significance of 3p26.3 deletion was unclear. Deletion of this region was previously described in patients with ID and atypical autism [46]; however, it was also reported in four generations of a family that were apparently healthy [47].

## 5. Conclusions

We detected subtelomeric rearrangements in 6.1% of the patients with ID. The sensitivity of subtelomeric FISH increases when preselection criteria were applied. We reported clinical entities observed in a patient with der(20)t(X;20)(p22.3;q13.3), a patient with der(3)t(X;3)(p22.3;p26.3), and a patient with der(10)t(7;10)(p22.3;q26.3). These rearrangements have never been or had rarely been reported in literature. Even though subtelomeric FISH is a very robust technique, as it can detect and locate rearrangements that involve small and specific regions of the chromosomes, uncover low level mosaicism, and identify balanced chromosomal rearrangements, CMA is currently recommended as a first-tier test and replaces the standard karyotype and subtelomeric FISH analyses for patients with ID of unknown causes [48]. However, in countries where CMA is unavailable or unaffordable, FISH is a useful technique to screen for subtelomeric rearrangements. In this study, we added information regarding clinical entities of our patients with subtelomeric rearrangements. This information requires accumulation from case reports and is useful for genetic counselling.

## Figures and Tables

**Table 1:** Clinical features of 5 patients with subtelomeric rearrangements.

Patient number	Patient 1	Patient 2	Patient 3	Patient 4	Patient 5
Age (mo)	60	96	180	36	72

Gender	Male	Female	Female	Male	Male

Karyotype	46,XY.ish del(4)(p16.3)(GS-36-P21−)	46,XX.ish del(1)(p36.32)(RP11-465B22−)dn	46,XX.ish der(10)t(7;10)(p22.3;q26.3)(GS-164-D18+;RP11-108K14−)	46,XY.ish der(20)t(X;20)(p22.3;q13.3)(RP13-465B17+;RP11-11M20−)	46,XY.ish der(3)t(X;3)(p22.3;p26.3)(RP13-465B17+;RP11-306H5−)dn

Interpretation	4p16.3 deletion	1p36.3 deletion	7p22.3 duplication and 10q26.3 deletion	Xp22.3 duplication and 20q13.3 deletion	Xp22.3 duplication and 3p26.3 deletion

Growth		Height and weight below the 3rd percentile	Height and weight below the 3rd percentile		

Head, face	Dolichocephaly, tall and prominent forehead	Triangular face with flat midface, prominent forehead	Microcephaly, low posterior hair line, webbed-neck		Dolichocephaly, prominent forehead, fair hair, low posterior hair line

Ocular region			Left esotropia	Mild hypertelorism	Upslanting eyebrows, mild hypertelorism, downslanting palpebral fissure

Nose	Broad nasal bridge				

Ears	Prominent ears with thick helix, bilaterally	Simple ears	Low set ears		

Mouth region		Cleft palate	Downturned corner of the mouth		Downturned corner of the mouth

Thorax, abdomen, extremities	Normal	Congenital heart defects including ventricular septal defect, patent ductus arteriosus, coarctation of the aorta	Cubitus valgus, simian crease on the right hand	Simian crease on both hands	Normal

Other anomalies	Sacral dimple				Shawl scrotum

Level of ID	IQ ~ 28–30 (severe)	IQ < 24 (severe)	IQ ~ 39–41 (moderate)	IQ = 42 (moderate)	IQ < 30 (severe)

Other tests	Abnormal EEG compatible with seizure disorder; normal hearing and thyroid function tests	Normal brain MRI; normal vision and hearing tests			Normal EEG and skull X-ray; normal hearing test

**Table 2:** Frequencies and details of subtelomeric rearrangements detected by FISH in patients with ID.

Reference	Total cases	Positive cases	Frequency (%)	De novo	Familial	Unknown inheritance
Knight et al., 1999 [4]	466^†^	22	4.7	*Deletion*: del(1)(p36.3)dn [*n* = 2], del(1)(q44)dn, del(6)(p25)dn, del(9)(p24)dn, del(13)(q34)dn, del(22)(q13.3)dn [*n* = 3] *Derivative*: der(9)t(9;16)(p24;q24)dn, der(13)t(Y;13)(p11.3;q34)dn, der(18)t(X;18)(q28;q23)dn	*Derivative*: der(12)t(6;12)(q27;p13.3)mat, der(4)t(4;22)(p16;q13.3)pat, der(9)t(9;13)(q34;p11.1)mat, der(1)t(1;13)(q44;q34)mat, der(4)t(4;11)(p16;p15.5)pat, der(4)t(4;6)(q35;q27)pat, der(1)t(1;19)(p36.3;q13.4)pat, der(4)t(4;20)(q35;p13)pat, der(8)t(8;20)(p23;p13)mat, der(7)t(2;7)(q37;q36)pat	

Joyce et al., 2001 [5]	213^a,†^	11	5.2	*Deletion*: del(1)(p36.2)dn, del(15)(q26.2)dn *Derivative*: der(13)t(13;19)(q34;q13.43)dn, der(4)t(4;8)(p16.1;p23.1)dn, der(4)t(4;11)(p16.3;p15.5)dn, mos der(18)t(4;18)(p16.3;q23)dn	*Deletion*: del(8)(p23.3)pat^*∗*^ *Derivative*: der(2)t(2;14)(q27.3;p11)pat, der(2)t(2;7)(q37.2;q36.3)mat, der(17)t(11;17)(p15.5;p13.3)mat, der(22)t(14;22)(q32.33;q13.31)pat	

Fan et al., 2001 [6]	150^‡^	6	4.0	*Deletion*: del(1)(ptel)dn [*n* = 2]	*Derivative*: der(1)t(1;3)(qtel;qtel)pat, der(18)t(7;18)(ptel;qtel)mat	*Derivative*: der(4)t(4;12)(ptel;qtel), der(5)t(5;20)(ptel;ptel)

Sismani et al., 2001 [7]	70^†^	1	1.4	*Deletion*: del(8)(pter)dn		

Anderlid et al., 2002 [8]	111^†^	11	9.9	*Deletion*: del(2)(qter)dn, del(4)(pter)dn, del(6)(qter)dn, del(9)(qter)dn *Derivative*: der(22)t(20;22)(qter;qter)dn	*Derivative*: der(4)t(2;4)(qter;qter)pat, der(21)t(9;21)(qter;qter)mat, der(17)t(12;17)(qter,qter)pat *Others*: rec(6)dup(6p)inv(6)(p23q27)pat [*n* = 2 siblings]	*Deletion*: del(22)(qter)

Baker et al., 2002 [9]	250^†^	8	3.2	*Deletion*: 22q− dn *Derivative*: der(5)t(5;16)(q35.3;q24.3)dn	*Deletion*: 20p− mat^*∗*^ *Derivative*: der(5)t(5;16)(q35.3;q24.3)pat, der(14)t(9;14)(q34.3;q32.33)mat *Others*: insertion (1p− and 9p+)pat	*Deletion*: 1p− *Others*: inversion (1p+ and 1q−)

Popp et al., 2002 [10]	30^‡^	6	20.0	*Derivative*: der(3)t(3;16)(p25;p13.2)dn, der(8)t(8;11)(p23.1;p15.5)dn, der(7)t(7;7)(p22;q36)dn	*Deletion*: del(8)(p23.1p23.1)pat^b^ *Derivative*: der(5)t(3;5)(q27;p15.3)pat, der(16)t(16;19)(p13.3;p13.3)mat	

Dawson et al., 2002 [11]	40^†^	3	7.5	*Deletion*: del(9)(q34.3)dn	*Derivative*: der(9)t(5;9)(q35.3;q34.3)pat	*Derivative*: der(3)t(3;11)(p36.3;p15.5)

Clarkson et al., 2002 [12]	50^†^	2	4.0		*Derivative*: der(5)t(5;21)(p13;q21.1)mat *Others*: rec(11)dup(11p)inv(11)(p15.5q24.3)pat	

van Karnebeek et al., 2002 [13]	184^†^	1	0.5	*Deletion*: del(12)(q24.33)dn		

Kirchhoff et al., 2004 [14]	94^‡^	3	3.2	*Deletion*: del(22)(qtel)dn [*n* = 2] *Derivative*: del(4)(qtel) and dup(7)(ptel) dn		

Font-Montgomery et al., 2004 [15]	43^‡^	6	14.0	*Deletion*: del(6)(q27)dn, del(9)(q34.3)dn *Derivative*: der(6)t(1;6)(q44;q27)dn, der(Y)dn (trisomy for PAR of Xp/Yp)	*Derivative*: der(4)t(4;5)(q35;p15.3)pat, der(2)t(2;17)(q37.3;p13)pat	

Walter et al., 2004 [16]	50^‡^	10	20.0	*Deletion*: del(22)(q13.32)dn, del(4)(p16.3)dn *Derivative*: der(7)t(7;9)(q36.3p24.1)dn, der(12)t(12;20)(p13p13.3)dn *Others*: t(17;20)(p13.3q13.33)dn	*Derivative*: der(4)t(4;7)(p16.3p22)pat, der(9)t(9;18)(p23q22.3)pat, der(7)t(7;10)(q36.3q26.3)mat, der(18)t(18;21)(q22.1q21.3)mat *Others*: der(7)inv(7)(p22q36.3)mat	

Rodriguez-Revenga et al., 2004 [17]	30^‡^	2	6.7	*Deletion*: del(1)(p36)dn	*Derivative*: der(13)t(1;13)(qter;qter)mat	

Bocian et al., 2004 [18]	83^c,†^	9	10.8	*Deletion*: del(4)(p16.1p16.3)dn *Derivative*: der(13)t(X;13)(q28;q34)dn	*Derivative*: der(13)t(4;13)(p16;q34)pat, der(2)t(2;7)(q37;q36)pat, der(4)t(4;21)(p16;q22)pat, der(6)t(4;6)(q35;q27)pat, der(4)t(4;7)(q33;q34)mat, der(10)t(10;19)(q26;p13.3)mat *Others*: rec(5)dup(5)(q35.3)inv(5)(p15.33q35.3)pat	

Velagaleti et al., 2005 [19]	18^†^	2	11.1			*Deletion*: del(2)(q27.3) *Others*: t(3;7)(q27;p21.2)

Baroncini et al., 2005 [20]	219^†^	12	5.5	*Deletion*: del(1pter)dn, del(7pter)dn, del(9pter)dn, del(9qter)dn, del(20pter)dn, del(22qter)dn *Derivative*: der(6)t(6;18)(qter;pter)dn, der(18)t(8;18)(pter;qter)dn	*Derivative*: der(12)t(12;17)(pter;qter)mat	*Derivative*: der(2)t(2;17)(qter;qter) *Others*: t(4;18)(pter;qter), t(1;16)(pter;pter)

Sogaard et al., 2005 [21]	132^†^	9	6.8	*Deletion*: del(1)(p36.3)dn, del(4)(p16.1)dn, del(5)(q35)dn *Derivative*: mos der(22)t(12;22)(p13;p?)dn	*Derivative*: der(13)t(5;13)(q35.2;q34)mat [*n* = 2 siblings], der(9)t(9;22)(q34.2;q13.3)pat	*Deletion*: del(2)(q37.2) *Derivative*: der(2)t(2;22)(q37.2;q1?)

Yu et al., 2005 [22]	534^†^	7	1.3	*Deletion*: del(9)(q34)dn *Derivative*: der(4)t(4;10)(q35.2;p15.3)dn	*Derivative*: der(22)t(4;22)(p16;q13)pat, der(10)t(10;11)(q26.1;q23.3)mat, der(X)t(X;4)(p22.3;q35.2)mat, der(10)t(7;10)(p22.3;q26.3)pat	*Deletion*: del(4)(q35)

Erjavec-Škerget et al., 2006 [23]	100^‡^	6	6.0	*Deletion*: del(X)(pter−)dn	*Derivative*: der(21)t(21;8)(qter−,qter+)pat, der(11)t(11;10)(qter−,qter+)pat	*Deletion*: del(9)(pter−)(23→pter−) *Derivative*: der(13)t(13;10)(qter−,q23.3→qter+) *Others*: rec(X)(qter+, pter−)

Palomares et al., 2006 [24]	50^‡^	5	10.0	*Deletion*: del(9)(q34.3)dn, del(2)(q37.3)dn *Derivative*: der(1)t(1;22)(p36.3;q13.3)dn, der(15)t(15;17)(q26.3;p25.3)dn	*Derivative*: der(2)t(2;10)(q37.3;q26.1)pat	

Rauch et al., 2006 [25]	500^†^	9	1.8	*Deletion*: del(1)(pter)dn, del(5)(qter)dn, del(9)(qter)dn, del(16)(pter)dn, del(22)(qter)dn *Others*: t(11;20)(pter;qter)dn	*Derivative*: der(14)t(14;20)(qter;qter)pat, der(10)t(9;10)(q34.1;q26.1)familial, der(10)t(10;22)(qter;qter)pat	

Ravnan et al., 2006 [26]	11688^†^	291^d^	2.5	*Deletion*: del(1)(pter)dn [*n* = 10], del(1)(qter)dn, del(2)(qter)dn, del(3)(qter)dn [*n* = 4], del(4)(pter)dn, del(4)(qter)dn, del(6)(qter)dn [*n* = 2], del(7)(pter)dn, del(8)(pter)dn, del(9)(pter)dn [*n* = 4], del(9)(qter)dn [*n* = 6], del(10)(pter)dn, del(10)(qter)dn [*n* = 2], del(13)(qter)dn, del(16)(pter)dn [*n* = 4], del(18)(qter)dn, del(20)(pter)dn [*n* = 3], del(20)(qter)dn [*n* = 2], del(22)(qter)dn [*n* = 2] *Derivative*: der(19)t(19;19)(pter;qter)dn, der(13)t(9;13)(qter;pter)dn, der(13)t(13;17)(pter;qter)dn, der(14)t(14;14)(pter;qter)dn, der(14)t(14;16)(pter;pter)dn, der(15)t(X;15)(qter;pter)dn, der(22)t(22;22)(pter;qter)dn, der(1)t(1;4)(pter;pter)dn, der(1)t(1;10)(pter;pter)dn, der(4)t(X;4)(qter;qter)dn, der(6)t(1;6)(qter;qter)dn, der(7)t(7;22)(qter;qter)dn, der(8)t(8;9)(pter;qter)dn, der(9)t(3;9)(pter;pter)dn, der(9)t(X;9)(qter;pter)dn, der(10)t(10;16)(pter;pter)dn, der(13)t(3;13)(qter;qter)dn, der(15)t(X;15)(qter;qter)dn, der(16)t(16;22)(pter;qter)dn, der(20)t(9;20)(pter;pter)dn, der(22)t(6;22)(pter;qter)dn, der(Y)t(Y;X or Y)(qter;pter)dn	*Deletion*: del(4)(qter)mat^*∗*^, del(11)(qter)mat^*∗*^ *Derivative*: der(7)t(7;16)(pter;pter)pat, der(10)t(10;16)(qter;pter)mat, der(15)t(15;19)(pter;qter)mat, der(2)t(2;17)(qter;pter)pat, der(2)t(2;20)(qter;qter)pat, der(2)t(2;22)(qter;qter)mat, der(4)t(3;4)(qter;pter)pat, der(4)t(4;5)(qter;pter)pat, der(5)t(2;5)(pter;pter)mat, der(5)t(5;7)(pter;pter)mat, der(5)t(5;20)(pter;pter)pat, der(6)t(6;21)(qter;qter)mat, der(7)t(7;19)(pter;qter)mat, der(7)t(5;7)(pter;qter)mat, der(7)t(7;8)(qter;pter)mat, der(7)t(7;11)(qter;pter)mat, der(8)t(2;8)(pter;pter)mat, der(9)t(9;16)(qter;pter)mat, der(10)t(1;10)(pter;qter)mat, der(10)t(8;10)(qter;qter)mat, der(10)t(10;17)(qter;qter)mat, der(11)t(2;11)(qter;qter)mat, der(12)t(12;17)(pter;pter)pat, der(12)t(12;19)(pter;qter)pat, der(12)t(12;19)(qter;pter)pat, der(17)t(9;17)(qter;pter)mat, der(18)t(4;18)(qter;qter)mat, der(21)t(5;21)(pter;qter)pat, der(22)t(16;22)(qter;qter)pat, der(X)t(X;X or Y)(pter;qter)mat^*∗*^	*Deletion*: del(1)(pter) [*n* = 18], del(2)(pter) [*n* = 2], del(2)(qter) [*n* = 6], del(3)(pter) [*n* = 4], del(3)(qter) [*n* = 4], del(4)(pter) [*n* = 7], del(4)(qter) [*n* = 4], del(4)(qter) and der(8)t(4;8)^e^, del(5)(pter), del(6)(pter), del(6)(qter) [*n* = 2], del(7)(pter), del(7)(qter), del(8)(pter) [*n* = 3], del(9)(pter), del(9)(qter) [*n* = 5], del(10)(pter) [*n* = 2], del(10)(qter) [*n* = 6], del(11)(qter) [*n* = 3], del(12)(pter), del(13)(qter) [*n* = 2], del(14)(qter) [*n* = 3], del(15)(qter), del(16)(pter), del(17)(pter) [*n* = 2], del(18)(qter) [*n* = 2], del(20)(pter) [*n* = 5], del(21)(qter), del(22)(qter) [*n* = 13], del(X)(pter), del(X)(qter), del(Y)(pter) [*n* = 2] *Derivative*: der(1)t(1;17)(pter;pter), der(10)t(10;16)(qter;pter), der(15)t(7;15)(qter;qter), der(19 or 20)t(X or Y;19 or 20)(qter;pter or qter), der(13)t(11;13)(qter;pter), der(14)t(7;14)(qter;pter), der(15)t(15;16)(pter;qter), der(15)t(X or Y;15)(qter;pter), der(21)t(17;21)(qter;pter), der(21)t(21;22)(pter;qter), der(22)t(1;22)(qter;pter), der(1)t(1;1)(pter;qter), der(1)t(1;10)(pter;pter), der(1)t(X or Y;1)(qter;pter), der(1)t(1;4)(qter;qter), der(1)t(1;3)(qter;qter), der(1)t(1;15)(qter;qter), der(1)t(1;18)(qter;pter), der(1)t(1;22)(qter;qter), der(2)t(1;2)(qter;qter), der(2)t(2;12)(qter;qter), der(4)t(4;6)(pter;pter) [*n* = 2], der(4)t(4;8)(pter;pter) [*n* = 2], der(4)t(4;11)(pter;pter), der(4)t(X or Y;4)(qter;qter), der(5)t(2;5)(pter;pter), der(5)t(5;9)(pter;pter), der(5)t(5;10)(pter;qter), der(5)t(5;14)(pter;qter), der(6)t(6;10)(pter;qter), der(6)t(6;12)(pter;qter), der(6)t(3;6)(qter;qter), der(6)t(6;7)(qter;pter), der(7)t(7;7)(pter;qter), der(7)t(7;16)(pter;pter), der(7)t(2;7)(qter;qter), der(7)t(7;9)(qter;qter), del(4)(qter) and der(8)t(4;8)^e^, der(8)t(8;8)(pter;qter), der(8)t(8;10)(pter;qter), der(8)t(8;12)(pter;pter) [*n* = 2], der(8)t(8;18)(pter;qter), der(9)t(7;9)(pter;qter), der(9)t(9;17)(qter;pter), der(10)t(7;10)(qter;pter), der(10)t(4;10)(pter;qter), der(10)t(4;10)(qter;qter), der(10)t(8;10)(qter;qter), der(10)t(10;10)(qter;pter), der(10)t(10;21)(qter;qter), der(11)t(11;12)(qter;qter) [*n* = 2], der(12)t(12;20)(pter;pter), der(13)t(2;13)(qter;qter), der(13)t(3;13)(qter;qter), der(14)t(X or Y;14)(pter;qter), der(15)t(3;15)(qter;qter), der(15)t(9;15)(qter;qter), der(16)t(16;16)(pter;qter), der(18)t(18;18)(pter;qter), der(18)t(2;18)(pter;qter), der(18)t(4;18)(qter;qter) [*n* = 2], der(18)t(10;18)(pter;qter), der(18)t(14;18)(qter;qter), der(20)t(5;20)(qter;pter), der(21)t(14;21)(qter;qter), der(22)t(12;22)(qter;qter), der(X)t(X;14)(pter;qter), der(X)t(X;X or Y)(pter;qter) [*n* = 2], der(X)t(X;3)(qter;pter), del(X)(pter)/der(X)t(X;15)(pter;qter) *Others*: dup(4)(qter), idic(Y)(q11.2), t(1;5)(qter;qter), t(6;12)(qter;qter), t(19;21)(pter;qter), t(5;6)(qter;qter), der(22)ins(22;X or Y)(q11.2;pter)

Ütine et al., 2009 [27]	130^†^	3	2.3	*Deletion*: del(22)(qter)dn *Derivative*: der(4)t(4;8)(pter;pter)dn	*Derivative*: der(9)t(4;9)(qter;pter)mat	

Mihçi et al., 2009 [28]	107^‡^	9	8.4	*Deletion*: del(1)(pter−)dn [*n* = 2], del(3)(qter−)dn, del(9)(pter−)dn, del(9)(qter−)dn *Derivative*: der(18)t(18;22)(qter−;qter+)dn	*Derivative*: der(5)t(5;15)(pter−;qter+)pat [*n* = 2 siblings] *Others*: rec(10)dup(10p)inv(10)(p13q26)mat	

Belligni et al., 2009 [29]	76^‡^	10	13.2	*Deletion*: del(1)(p36)dn [*n* = 2], del(9)(q34qter)dn *Derivative*: der(6)(ptel−;qtel++)dn, der(5)t(5pter;10qter)dn, t(1;13)(p32.2;q31.1)dn^f^	*Derivative*: der(9)t(9;16)(9pter-9q34.3::16q24.3-qter)pat, der(20)t(16;20)(q24;q13.3)pat, der(6)t(6;1)(p22.3;q44)mat, der(7)t(7;12)(q34;q24.32)mat	

Bogdanowicz et al., 2010 [30]	76^‡^	4	5.3	*Deletion*: del(1)(p36.3−)dn [*n* = 2] *Derivative*: der(1)t(1;12)(p36.3,q24.3+)dn *Others*: t(19;22)(q13.4−,q13.3+;q13.3−,q13.4+)dn		

dos Santos and Freire-Maia, 2012 [31]	15^‡^	0	0			

Our study	82^†^	5	6.1	*Deletion*: del(1)(p36.32)dn *Derivative*: der(3)t(X;3)(p22.3;p26.3)dn		*Deletion*: del(4)(p16.3) *Derivative*: der(10)t(7;10)(p22.3;q26.3), der(20)t(X;20)(p22.3;q13.3)

*Summary *	*15,591*	*473*	*3.0*			

^a^We included 13 patients, who had subtle subtelomeric rearrangements detected by high resolution G-banding. Of these, 10 cases had subtelomeric rearrangements detected by FISH. We excluded del(4)(q35.2)dn and der(Y)t(Y;17)(pter;q25.3)dn that were detected in 150 controls. ^b^Father was balanced translocation with deletion and duplication of 8p231. ^c^We excluded one case as FISH was performed in a normal father of 3 deceased patients with severe ID. The FISH result in the father revealed t(7;10)(q36;q26). ^d^We excluded 9 patients with interstitial deletions that were detected with control probes. ^e^The same individual. ^f^Karyotype nomenclature here is as shown in the original article but it was actually an unbalanced rearrangement (trisomy for 1p32.2 and monosomy for 13q31.1). Note that 1p32.1 and 13q31.1 are actually not subtelomeric regions. ^*∗*^The same abnormality also found in parent but not a familial variant. ^†^ID with or without dysmorphism. ^‡^ID with dysmorphism. +: gain, −: loss, dn: de novo, del: deletion, der: derivative, dim: partial deletion or diminished signal, dup: duplication, ins: insertion, inv: inversion, idic: isodicentric, mat: maternal, mos: mosaic, pat: paternal, rec: recombinant, tel: telomere, ter: terminal, and t: translocation.

**Table 3:** Familial variants and possible variants detected by FISH in patients with ID.

Reference	Number of cases	Variant
Fan et al., 2001 [6]	8	del(2)(qter) [*n* = 8]

Anderlid et al., 2002 [8]	2	del(2)(qter) [*n* = 2]

Baker et al., 2002 [9]	5	del(12)(pter) [*n* = 1], del(2)(qter) [*n* = 4]

Dawson et al., 2002 [11]	1	del(Y)(qter) [*n* = 1]

Clarkson et al., 2002 [12]	1	del(2)(qter) [*n* = 1]

van Karnebeek et al., 2002 [13]	11	del(2)(qter) [*n* = 7], del(X)(pter) [*n* = 3], del(Y)(pter) [*n* = 1]

Kirchhoff et al., 2004 [14]	5	del(2)(qter) [*n* = 5]

Ravnan et al., 2006 [26]	56	del(3)(pter) [*n* = 1], del(4)(qter) [*n* = 2], del(10)(qter) [*n* = 1], del(17)(pter) [*n* = 1], del(20)(pter) [*n* = 1], del(21)(qter) [*n* = 2], del(Y)(qter) [*n* = 10], dim(4)(pter) [*n* = 1], dim(10)(qter) [*n* = 14^*∗*^], dim(14)(qter) [*n* = 3], dim(Y)(pter) [*n* = 1], dup(8)(pter) [*n* = 1], ?dup(10)(qter) [*n* = 1], mos dup(10)(qter) [*n* = 1], der(3)t(3;14)(pter;qter) [*n* = 1], der(18)t(16;18)(qter;pter) [*n* = 5], der(18)t(17;18)(pter;pter) [*n* = 1], der(20)t(X or Y;20)(qter;pter) [*n* = 1], der(X)t(X;X or Y)(qter;pter) [*n* = 5], der(22)t(4;22)(pter;pter) [*n* = 3]

Erjavec-Škerget et al., 2006 [23]	5	del(2)(qter) [*n* = 5^*∗∗*^]

Rauch et al., 2006 [25]	52	del(9)(pter) [*n* = 1], del(Y)(qter) [*n* = 1], del(2)(qter) [*n* = 5], dim(2)(qter) [*n* = 24], dim(4)(qter) [*n* = 18], dim(2qter and 4qter) [*n* = 1], dim(15)(qter) [*n* = 2]

Bogdanowicz et al., 2010 [30]	3	del(2)(qter) [*n* = 2^*∗∗∗*^], dup(7)(qter) [*n* = 1]

*Total*	*149*	

^*∗*^One patient had dim(10)(qter) familial variant and a clinically significant del(16)(pter). ^*∗∗*^One patient had del(2)(qter) familial variant and a clinically significant del(X)(pter). ^*∗∗∗*^One patient had del(2)(qter) familial variant and a clinically significant del(1)(pter).
